# Establishment and Analysis of a Combined Diagnostic Model of Polycystic Ovary Syndrome with Random Forest and Artificial Neural Network

**DOI:** 10.1155/2020/2613091

**Published:** 2020-08-20

**Authors:** Ning-Ning Xie, Fang-Fang Wang, Jue Zhou, Chang Liu, Fan Qu

**Affiliations:** ^1^Women's Hospital, School of Medicine, Zhejiang University, Hangzhou 310006, China; ^2^College of Food Science and Biotechnology, Zhejiang Gongshang University, Hangzhou 310018, China; ^3^Zhejiang Chinese Medical University, Hangzhou 310053, China

## Abstract

Polycystic ovary syndrome (PCOS) is one of the most common metabolic and reproductive endocrinopathies. However, few studies have tried to develop a diagnostic model based on gene biomarkers. In this study, we applied a computational method by combining two machine learning algorithms, including random forest (RF) and artificial neural network (ANN), to identify gene biomarkers and construct diagnostic model. We collected gene expression data from Gene Expression Omnibus (GEO) database containing 76 PCOS samples and 57 normal samples; five datasets were utilized, including one dataset for screening differentially expressed genes (DEGs), two training datasets, and two validation datasets. Firstly, based on RF, 12 key genes in 264 DEGs were identified to be vital for classification of PCOS and normal samples. Moreover, the weights of these key genes were calculated using ANN with microarray and RNA-seq training dataset, respectively. Furthermore, the diagnostic models for two types of datasets were developed and named neuralPCOS. Finally, two validation datasets were used to test and compare the performance of neuralPCOS with other two set of marker genes by area under curve (AUC). Our model achieved an AUC of 0.7273 in microarray dataset, and 0.6488 in RNA-seq dataset. To conclude, we uncovered gene biomarkers and developed a novel diagnostic model of PCOS, which would be helpful for diagnosis.

## 1. Introduction

Polycystic ovary syndrome (PCOS), as a heterogeneous endocrine disorder, is closely associated with menstrual dysfunction, infertility, hirsutism, acne, obesity, and metabolic syndrome [1]. The three major diagnostic criteria of PCOS widely followed are criteria raised by National Institutes of Health (NIH) [2], 2003 Rotterdam Consensus raised by European Society of Human Reproduction and Embryology (ESHRE) and American Society for Reproductive Medicine (ASRM) [3, 4], and criteria raised by Androgen Excess Society (AES) [5]. However, these criteria have created some controversy in the field [6]. The multifactorial etiology of PCOS is underpinned by a complex genetic architecture [7]. Ethnicity is eminently related to PCOS phenotype because of the different genetic and environmental propensity to metabolic disorders [8–10].

Although the identified genetic risk markers can be used as predictive and diagnostic tools for PCOS, they may not possess the strong power due to the complicated genetic architecture [6]. Combination of various markers in diagnostic panels may significantly improve the success [11]. Many studies have successfully used genetic risk scores to explain increasing amounts of variance in diseases [12].

In recent years, the wide application of microarray technology and more advanced, accurate RNA-sequencing technology made the study of disease mechanism more convenient. In view of the differences between the two platforms, it is necessary to analyze the data of the two platforms separately.

The main difficulty arisen in establishing a classification model using gene expression data was how to find the most meaningful index or feature for classification. To address this, various machine learning approaches such as random forest (RF) [13, 14] and artificial neural network (ANN) [15] were utilized. The single or combined use of these algorithms has contributed much in gene expression data classification [16], disease diagnosis [17], cell migration [18], and microbiome research [19]. Given their high classification accuracy and convenience, they have become powerful tools to learn feature representations.

In this work, we established a diagnosis model of PCOS using microarray and RNA-seq data from Gene Expression Omnibus (GEO) database with the combined utilization of RF and ANN. Firstly, the RF classifier was used to identify the key genes for classification, and then, the ANN was performed to calculate the weights of the key genes in microarray and RNA-seq data, respectively. Finally, a scoring model named neuralPCOS was developed with the integration of RF and ANN. To validate the accuracy and superiority of the diagnosis model we established, we evaluated the performance with microarray and RNA-seq data and compared them to other marker genes obtained in previous studies [20, 21].

## 2. Materials and Methods

### 2.1. Study Design

**Figure 1:**
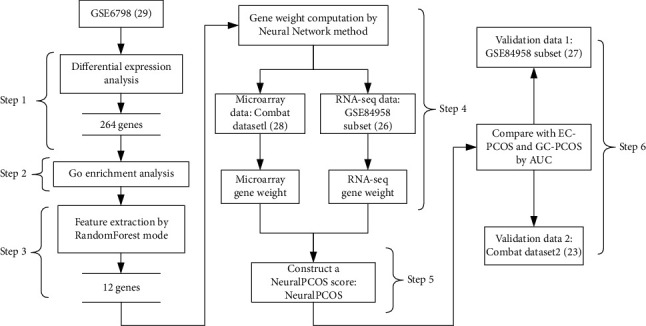
Schematic illustration of study design. A total of 264 differentially expressed genes (DEGs) were obtained in differential expression analysis with GSE6798 dataset (skeletal muscle, *n* = 29) (step 1), and functional enrichment analysis were also performed (step 2). All the 264 DEGs were tested for their potential as classification-related genes with random forest model, and 12 key genes were identified (step 3). Artificial neural network (ANN), another machine learning algorithm, was used to calculate the weight of genes (step 4). Therefore, a versatile classification model, designated as neuralPCOS, was established with the use of RF and ANN (step 5). Finally, the utility of neuralPCOS was validated in microarray data and RNA-seq data (step 6).

For establishment of the diagnostic model of PCOS, RF and ANN were adopted in this study. The study overview was schematically depicted in Figure 1. GSE6798 dataset (*n* = 29) was used for the differentially expressed genes (DEGs) screening (step 1). Gene ontology (GO) enrichment analysis (step 2) and the acquisition of key genes for classification by RF (step 3) were further performed. After computing the gene weight using ANN in two kinds of expression data (microarray and RNA-seq) (step 4), a classification model was developed (step 5). Finally, we used two independent dataset (the microarray ComBat dataset2 and the RNA-seq–based dataset GSE84958) for further validation (step 6).

### 2.2. Data Selection and Preprocessing

In the present study, a wide search through the National Center for Biotechnology Information Gene Expression Omnibus database (NCBIGEO) platform was conducted with the key words “PCOS, human”. As shown in Table 1, 6 sets of microarray data and 1 set of RNA-seq data were downloaded from GEO database. In order to obtain one training dataset (microarray ComBat dataset1) with large sample size, three microarray datasets with small sample size (GSE137684, GSE137354, and GSE34526) were combined. Meanwhile, GSE43264 and GSE124226 were combined to form one validation dataset (microarray ComBat dataset2). These datasets were converted to logarithmic form after standardization, and the R package ComBat was used to remove the batch effects [22]. Two microarray datasets with 28 and 23 samples were obtained using classical and Bayesian correction methods.

### 2.3. Differentially Expressed Genes (DEGs) Screening

The dataset GSE6798, based on Affymetrix Human Genome U133 Plus 2.0 Array (Affymetrix Inc., Santa Clara, California, USA) contained 16 cases of PCOS and 13 cases of control, was used for DEGs analysis. The boxplot was performed using R package stats (v 3.5.0). The R package limma was used to calculate the DEGs between the PCOS and control samples by the classical Bayesian method with *P* < 0.01 and |logFoldchange| >0.26 [23] and was visualized by volcano plot [24].

### 2.4. Gene Ontology (GO) Enrichment Analysis

To further reveal the biofunction of selected DEGs, GO enrichment analysis, including biological process (BP), cellular component (CC), and molecular function (MF), was performed using R package clusterProfiler [25]. Significant enrichment terms were screened with the threshold adjusted *P* < 0.01 after adjusted by the Benjamini and Hochberg method. To eliminate some redundancies, GO terms that intersects more than 75% of the genes contained in term were removed. GObubble and GOChord were performed with R package GOplot to illustrate the functional analysis data [26].

### 2.5. Random Forest (RF) Classification

We used random forest to classify the DEGs with the R package randomforest [27]. Firstly, the optimal number of variables (mtry parameter, the optimal number of variables used in the binary tree in the specified node) was identified. All possible variables (1~2000) were looped into the random forest classifier. Each error rate was calculated, and the optimal number of variables was selected. Next, each error rate of 1~3000 trees was calculated, and the optimal tree number was determined by the lowest error rate and best stability. Based on the above-selected parameters, the random forest classifier was used to calculate the results, and the important genes were selected as the candidate PCOS-specific genes according to the Gini coefficient method.

### 2.6. Calculation of DEGs Weight by Artificial Neural Network (ANN)

The GSE84958 dataset was randomly divided into training data (*n* = 26) and validation data (*n* = 27). The RNA-seq training data GSE84958 (*n* = 26) and microarray ComBat dataset1 (*n* = 28) were used to construct the neural network model. The R package neuralnet was used for neural network analysis [28]. First of all, the integration data were filtered and normalized by min-max normalization. Secondly, the processed training data was inputted into the neural network model. Eleven genes were inputted and 3 hidden layers, and 2 outputs (normal and PCOS) were set in both microarray data and RNA-seq data. Finally, the output of the first hidden layer (input of the last output layer) in the network results were considered as the results of gene weight.

### 2.7. Neural-PCOS

We constructed an equation named neuralPCOS that could estimate the classification score of each gene in microarray data or RNA-seq data.

(1) $$\begin{array}{c} \text{neuralPCOS}=\sum \left(GeneExpression\times NeuralNetworkWeight\right). \end{array}$$

The gene expression value was multiplied by the weight of gene, and the results of all genes were added. (Note: before calculating the score, the expression data after log2 processing needs to be normalized by min-max normalization.)

### 2.8. Evaluation of Performance by Area under Curve (AUC)

The AUCs of three kinds of scores (neuralPCOS, EC-PCOS, GC-PCOS) were calculated in GSE84958 RNA-seq validation data (*n* = 27) and microarray validation data (*n* = 23) with R package pROC, respectively [29].

Three kinds of score:

1. neuralPCOS
2. EC-PCOS: three upregulated genes including insulin-like growth factor 1 (*IGF1*), phosphatase and tensin homolog (*PTEN*), and insulin-like growth factor-binding protein 1 (*IGFBP1*) in endometrial cells (ECs) of PCOS [20].
3. GC-PCOS: upegulated genes including hydroxy-delta-5-steroid dehydrogenase, 3 beta- and steroid delta-isomerase 2 (*HSD3B2*), steroidogenic acute regulatory protein (*STAR*), inhibin subunit beta A (*INHBA*), and cytochrome P450 family 19 subfamily A member 1 (*CYP19A1*) in granulosa cells (GCs) of PCOS [21].

## 3. Results

### 3.1. Identification of DEGs

**Figure 2:**
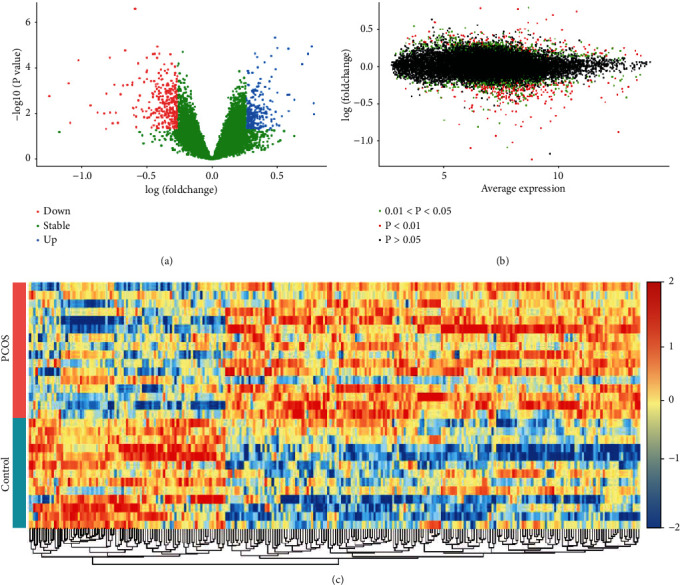
Analyses of DEGs in GSE6798 dataset (skeletal muscle, *n* = 29). (a) Volcano plot of differential gene expression with log (foldchange) as the abscissa and -log10 (*P* value) as the ordinate. Blue and red splashes represent the genes that were significantly up- or downregulated in PCOS, respectively. Green splashes mean genes without significantly different expression. *P* < 0.01, |logFC| >0.26. (b) Volcano plot of gene average expression level. The *x*-axis represents the average expression levels of genes in all samples. The *y*-axis indicates logFC. The red spots are DEGs with *P* < 0.01, the green spots, DEGs with 0.01 < *P* < 0.05; and the black spots, stable genes (*P* > 0.05). (c) Heatmap of the 264 DEGs in GSE6798 dataset. Each row represents a sample and each column represents a gene. Red color means a higher expression level; blue color means a lower expression level.

Firstly, the boxplot presented RNA expression level in GSE6798 (*n* = 29) (Figure S1). A total of 20174 gene symbols were identified after annotation, and the distribution of DEGs (*P* < 0.01, |logFC| >0.26) were represented by volcano plot, including 134 upregulated genes and 210 downregulated genes (Figure 2(a)). The volcano plot of gene average expression level was shown in Figure 2(b). Moreover, the genes with low expression level (last 25%) were removed, and 264 genes (*P* < 0.01, |logFC| >0.26) were obtained (Table S1). The heat map of the screened 264 DEGs in GSE6798 dataset was shown in Figure 2(c).

### 3.2. Functional Characterization of Selected DEGs

**Figure 3:**
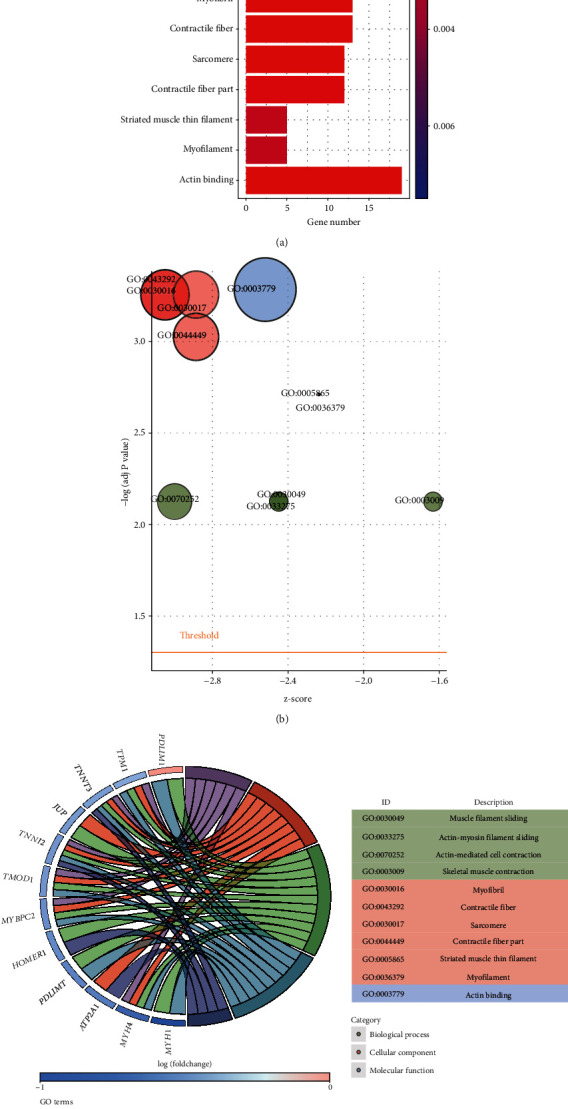
Gene ontology (GO) enrichment analysis. (a) The bar plot of enriched GO terms in biological process (BP), cell components (CC), and molecular function (MF). The *x*-axis represents the GOid, and *y*-axis represents the significance of terms. The terms are placed according to their z-score (which indicates that the term is more likely to increase or decrease). (b) The bubble plot of GO analysis of 264 DEGs. The z-score is assigned to *x*-axis and the negative logarithm of the adjusted *P* value to *y*-axis. Bubble size is proportional to the number of genes in GO terms, and the color represents three categories (green: BP; red: CC; blue: MF). (c) GOChord plot: a plot indicates the relationship between DEGs and their associated terms. The color represents upregulation (red) or downregulation (blue).

GO enrichment analysis for the selected 264 DEGs was carried out to identify the significantly enriched GO terms. The GObar showed the predominant significantly enriched GO terms (adjusted *P* < 0.01) (Figure 3(a)). Muscle filament sliding (adjusted *P* = 7.49*E* − 03), myofibril (adjusted *P* = 5.55*E* − 04), and actin binding (adjusted *P* = 5.19*E* − 04) were the most significantly enriched GO terms in BP, CC, and MF, respectively (Table S2). The 11 enriched terms were displayed in bubble plot (Figure 3(b)). The analysis revealed that skeletal muscle contraction was the most upregulated term; contractile fiber was the most downregulated one. After de-redundant the resulting GO terms, 5 enriched terms were obtained. To add quantitative molecular data in the GO terms of interest, GOChord was performed. It indicated that 12 DEGs were enriched into 5 Go terms, among which myofibril contained the most DEGs (Figure 3(c)).

### 3.3. Screening Candidate PCOS-Specific Genes by Random Forest

**Figure 4:**
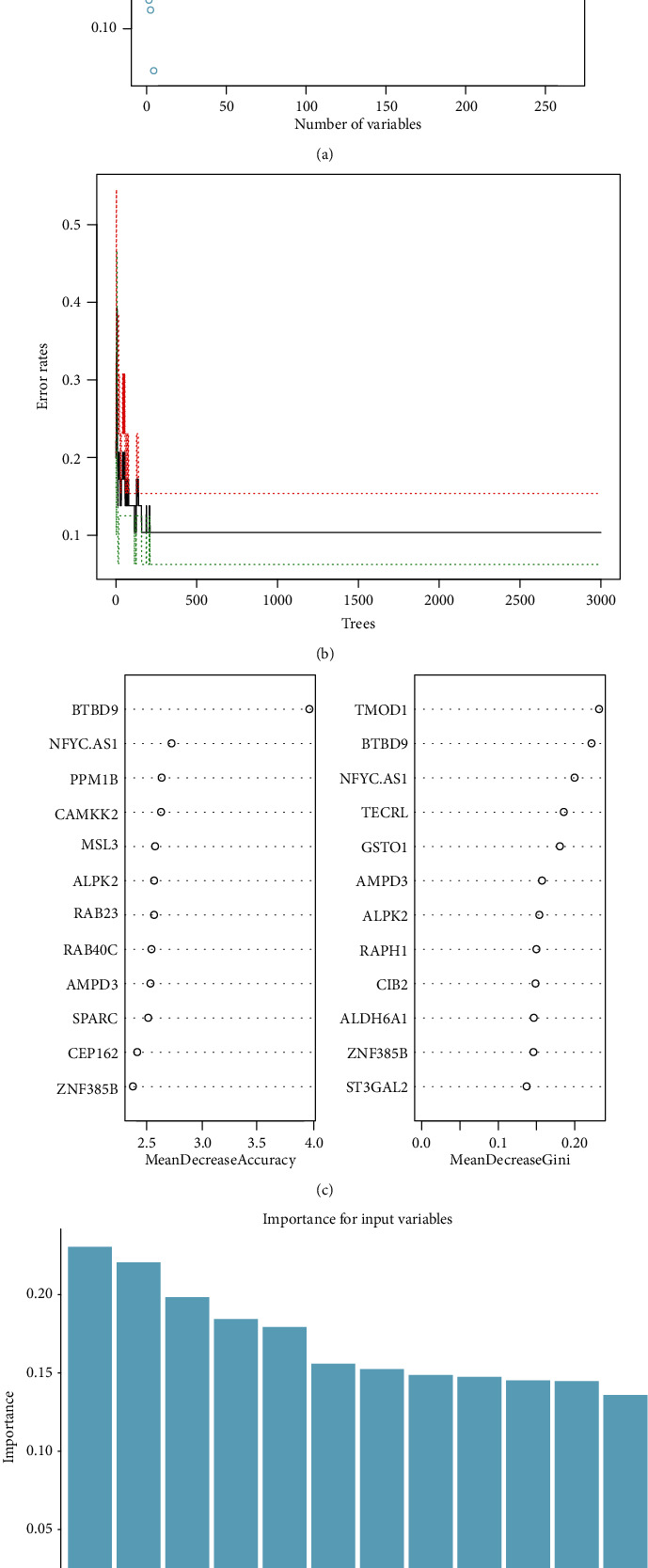
Screening candidate PCOS-specific genes by random forest. (a) Parameter selection for random forest classifier. The scatter plot of the variables and corresponding error rate. The *x*-axis is the number of variables, and the *y*-axis is the error rate. (b) The influence of the number of decision trees on the error rate. The *x*-axis is the number of decision trees and the *y*-axis is the error rate. (c) Ranking of input variables in the random forest model to classify PCOS and normal samples. Top 12 key genes were listed from the most important ones to the least ones based on MeanDecreaseAccuracy and MeanDecreaseGini. (d) Top 12 key genes in MeanDecreaseGini. The *x*-axis represents the genes, and the *y*-axis is the importance index.

In order to obtain more reliable PCOS-specific genes, we inputted the above 264 DEGs into the RF classifier. The lowest error rate occurred when the number of variables was 4 (Figure 4(a)); meanwhile, the optimal number of trees in RF classifier was set to 1000 due to the low error rate and stability (Figure 4(b)). Therefore, we finally choose 4 and 1000 trees as the final parameter in RF classifier to obtain the dimensional importance of all variables. Top 12 genes in the results of MeanDecreaseAccuracy and MeanDecreaseGini were shown in Figure 4(c). Finally, we selected 0.15 as the screening threshold of importance in MeanDecreaseGini result, and a set of 12 PCOS-specific DEGs was identified.

### 3.4. ANN Analysis

**Figure 5:**
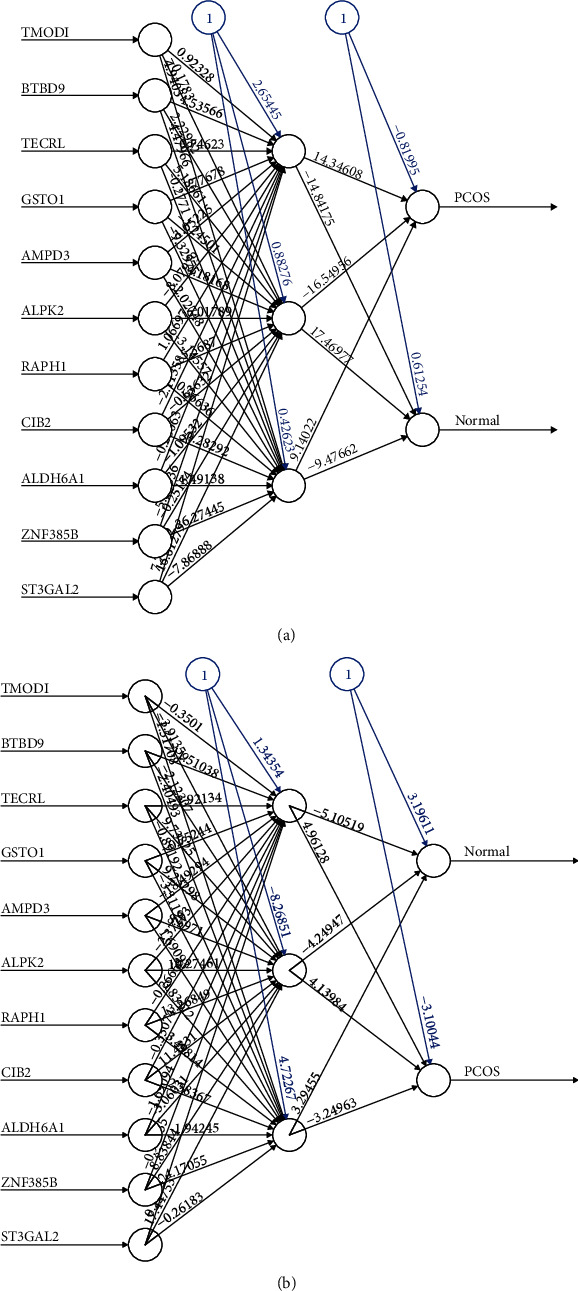
Neural network topology of two kinds of training data. (a) Neural network topology of microarray ComBat dataset1 (GSE137684, GSE137354, and GSE34526, *n* = 28) with 11 input layer, 3 hidden layer, and 2 output layer. (b) Neural network topology of RNA-seq data GSE84958 (adipose, *n* = 26) with 11 input layer, 3 hidden layer, and 2 output layer.

RF classifier identified the key genes, which optimally differentiated between PCOS and controls. To further construct a PCOS-specific scoring model, ANN analysis was performed to calculate the weight of 12 genes. Here, two parallel training processes were carried out according to format of the training data, including RNA-seq training data GSE84958 (*n* = 26) and microarray ComBat dataset1 (*n* = 28). ANN topology of microarray ComBat dataset1 and RNA-seq data indicated 11 input layer, 3 hidden layer, and 2 output layer (Figure 5). The weight of each gene was detailed in Table S3 for microarray ComBat dataset1 and Table S4 for RNA-seq data. Based above, we constructed a model for classifying the gene expression data between PCOS and control samples.

### 3.5. The Validation of neuralPCOS

**Figure 6:**
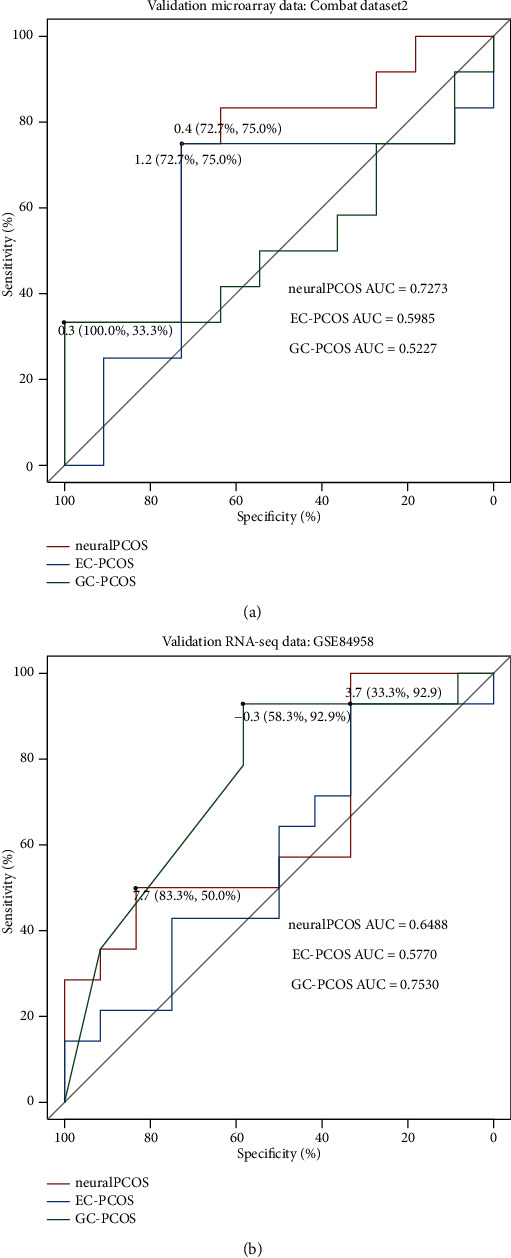
Performance evaluation of different classification models by the area under the receiver operating characteristic (ROC) curves and their AUC values. (a) In microarray ComBat dataset2 (GSE43264 and GSE124226, *n* = 23), neuralPCOS achieved superior performance (AUC: 0.7273), compared to the other two methods: EC-PCOS (AUC: 0.5985) and GC-PCOS (AUC: 0.5227). (b) In GSE84958 RNA-seq validation data (adipose, *n* = 27), neuralPCOS achieved an AUC of 0.6488, EC-PCOS (AUC: 0.5770) and GC-PCOS (AUC: 0.7530). The optimal threshold values were labeled at inflection points, and the sensitivities and specificities were listed in the bracket.

Microarray ComBat dataset2 (*n* = 23) and GSE84958 RNA-seq verification data (*n* = 27) were used to test the ability for classifying the samples in 3 classification models, including neuralPCOS constructed in this study and EC-PCOS and GC-PCOS from other researches. The performance of these models was examined using area under the receiver operating characteristic curve (ROC) (Figure 6). First, we estimated differences in the AUC values among 3 models in microarray data (Figure 6(a)). The results showed that neuralPCOS had a high-level classification performance with an AUC of 0.7273, compared with the AUC of EC-PCOS (0.5985) and GC-PCOS (0.5227). The optimal threshold values for 3 models were 1.2, 0.4, and 0.3, respectively. NeuralPCOS and EC-PCOS achieved the highest level of specificity (75.0%), and GC-PCOS had 100% sensitivity at optimal threshold value. The result of RNA-seq validation data suggested that the AUC score of neuralPCOS (0.6488) was higher than EC-PCOS (0.5770), but lower than GC-PCOS (0.7530). The optimal threshold values for 3 models were 7.7, 3.7, and -0.3, respectively. NeuralPCOS had the highest level of sensitivity than EC-PCOS and GC-PCOS (Figure 6(b)). From the above results, it can be concluded that the classification model established in this study was more suitable in microarray data than in RNA-seq data.

## 4. Discussion

In recent years, the development of machine learning algorithms and the availability of gene expression data in public databases provide approaches to infer biomarkers for disease diagnosis or prognosis in a wide range of fields [30–33]. In the field of PCOS, some attempts have been made to explore a better way for PCOS diagnosis by using various machine learning algorithms [34–38], among which, suitable algorithms using some clinical data, such as survey data [35] or pelvic ultrasound data, were used [37]. An algorithm was ever constructed to predict new PCOS candidates using the data from Polycystic Ovary Syndrome Database (PCOSDB; http://www.pcosdb.net/) [39] and the KnowledgeBase on Polycystic Ovary Syndrome (PCOSKB; http://pcoskb.bicnirrh.res.in) [36, 40]. Another study converted the ovary microarray data of GEO database to the gene set regularity (GSR) indices, and the GSR indices were then computed by the modified differential rank conversion algorithm [38]. Comparing with these studies, we aimed to develop a diagnostic model based on gene expression data using as many samples as possible from GEO database. We finally integrated RF and ANN algorithms to infer the key classification genes and calculate the weights of these genes.

In the present study, when identifying DEGs with GSE6798 dataset, we removed the DEGs with low expression level, which can obtain more authentic genes. GO enrichment analysis was performed and displayed by bar plot and bubble plot. Among the 11 enriched GO terms, 4 terms including actin binding [41], myofibril [42], sarcomere [42], and contractile fiber part [42] were also identified in other PCOS researches. We listed the top 12 core genes screened by the RF model for classification in DEGs based on MeanDecreaseGini. Moreover, 10 of the 12 genes were also regarded as PCOS candidate genes in other studies: tropomodulin 1 (*TMOD1*) [43]; BTB domain containing 9 (*BTBD9*) [44]; trans-2,3-enoyl-CoA reductase like (*TECRL*) [44, 45]; glutathione S-transferase omega 1 (*GSTO1*) [44, 46, 47]; adenosine monophosphate deaminase 3 (*AMPD3*) [45]; alpha kinase 2 (*ALPK2*) [48]; Ras association (RalGDS/AF-6) and pleckstrin homology domains 1 (*RAPH1*) [44, 45, 48, 49]; aldehyde dehydrogenase 6 family member A1 (*ALDH6A1*) [44, 45, 50–52]; zinc finger protein 385B (*ZNF385B*) [53]; ST3 Beta-galactoside alpha-2,3-sialyltransferase 2 (*ST3GAL2*) [44]. Given that RNA-seq technology has the superiorities to detect novel transcripts with wider dynamic range, higher specificity, and higher sensitivity than microarray technology [54], the gene expression data obtained by these two technologies may have some differences. In the study, we calculated the weights of core genes by ANN using each type of data separately. Although the weights of only 11 genes in both microarray data and RNA-seq data were calculated, 10 genes were verified in previous studies in both platforms. The novelty of our diagnostic model was that the scoring model was obtained by comprehensively considering the genes those are vital to classification and their weights. In order to validate the applicability and superiority of this model in different types of data, AUC analysis was performed in microarray ComBat dataset2 (*n* = 23) and RNA-seq validation dataset (GSE84958, *n* = 27). In the meanwhile, two sets of marker genes in other researches were also evaluated. One set of genes was the upregulated genes that involved in the insulin signaling pathway (*IGF1*, *PTEN*, and *IGFBP1*) [20]; the other was the upregulated genes including *HSD3B2*, *STAR*, *INHBA*, and *CYP19A1* [21]. The results of AUC scores indicated that our model achieved a superior performance compared with the other two sets of genes in microarray data, and moderate performance but highest level of sensitivity in RNA-seq data. Our model got high AUC scores, indicating it could separate PCOS samples from normal samples with a good probability in microarray data.

Even so, our study still has some limitations. Although our total sample size is not too small (PCOS: *n* = 76; normal: *n* = 57), the number of sample size in each dataset is small, and the individuals in integrated microarray training dataset are from different countries. To get microarray training and validation datasets with larger sample size, 3 and 2 small sample size datasets were combined, respectively. Although the batch effect was removed, it was still not the most suitable datasets. Another drawback of our study is that the expression data are from diverse tissues containing skeletal muscle, adipose, endometrium, and granulosa cells. Last but not least, we did not perform 10 fold cross-validation in ANN analyse due to the limited sample size. Although this is a compromising strategy in the case of limited sample size, our model has an excellent classification performance, a diagnostic model for single tissue type still needs to be constructed with more convincing datasets and machine learning algorithms in the future.

## 5. Conclusions

A novel diagnostic model for PCOS was established based on machine learning algorithms using microarray and RNA-seq datasets, which showed better prediction performance in microarray data than using existing marker genes.

## Figures and Tables

**Table 1:** Gene expression data from Gene Expression Omnibus (GEO) database.

Dataset ID	Total samples	Control	PCOS	Data type	Tissue type	Country
GSE6798	29	13	16	Microarray	Skeletal muscle	Denmark
GSE43264	15	7	8	Microarray	Adipose	Ireland
GSE34526	10	3	7	Microarray	Granulosa cells	India
GSE137684	12	4	8	Microarray	Granulosa cells	China
GSE137354	6	3	3	Microarray	Endometrium	China
GSE124226	8	4	4	Microarray	Adipose stem cells	USA
GSE84958	53	23	30	RNA-seq	Adipose	UK

## Data Availability

The data used to support the findings of this study are available from the corresponding author upon request.

## Supplementary Materials

Supplementary 1 Figure S1: boxplot for gene expression data in GSE6798 dataset. The abscissa axis indicates 29 samples in GSE6798 dataset. Axis of ordinates represents gene expression level.

Supplementary 2 Table S1: the selected 264 differentially expressed genes (DEGs) in GSE6798 dataset.

Supplementary 3 Table S2: significantly enriched gene ontology (GO) terms in biological process (BP), cell components (CC), and molecular function (MF).

Supplementary 4 Table S3: the weight of each gene for microarray ComBat dataset1.

Supplementary 5 Table S4: the weight of each gene for RNA-seq data.

## References

[1] Norman R. J., Dewailly D., Legro R. S., Hickey T. E. (2007). Polycystic ovary syndrome. *Lancet*.

[2] Zawadzki J. K., Dunaif A. (1992). Diagnostic criteria for polycystic ovary syndrome: towards a rational approach. *Polycystic Ovary Syndrome*.

[3] The Rotterdam ESHRE/ASRM-Sponsored PCOS Consensus Workshop Group (2004). Revised 2003 consensus on diagnostic criteria and long-term health risks related to polycystic ovary syndrome. *Fertility and Sterility*.

[4] The Rotterdam ESHRE/ASRM-Sponsored PCOS Consensus Workshop Group (2004). Revised 2003 consensus on diagnostic criteria and long-term health risks related to polycystic ovary syndrome (PCOS). *Human Reproduction*.

[5] Azziz R., Carmina E., Dewailly D. (2009). The Androgen Excess and PCOS Society criteria for the polycystic ovary syndrome: the complete task force report. *Fertility and Sterility*.

[6] Fauser B. C. J. M., Tarlatzis B. C., Rebar R. W. (2012). Consensus on women’s health aspects of polycystic ovary syndrome (PCOS): the Amsterdam ESHRE/ASRM-Sponsored 3rd PCOS Consensus Workshop Group. *Fertility and Sterility*.

[7] Jones M. R., Goodarzi M. O. (2016). Genetic determinants of polycystic ovary syndrome: progress and future directions. *Fertility and Sterility*.

[8] Sirota I., Stein D. E., Vega M., Keltz M. D. (2013). Increased insulin-resistance and beta-cell function in polycystic ovary syndrome women-does ethnicity play a role?. *Reproductive Sciences*.

[9] Louwers Y., Lao O., Kayser M. (2013). Inferred genetic ancestry versus reported ethnicity in polycystic ovary syndrome (PCOS). *Human Reproduction*.

[10] Azziz R., Ezeh U., Pall M., Dumesic D. A., Goodarzi M. O. (2010). Effect of race on the metabolic dysfunction of Polycystic Ovary Syndrome (PCOS): comparing African-American (AA) and Non-Hispanic White (NHW) patients.. *Endocrine Reviews*.

[11] Vilhjálmsson B. J., Yang J., Finucane H. K. (2015). Modeling linkage disequilibrium increases accuracy of polygenic risk scores. *The American Journal of Human Genetics*.

[12] Talmud P. J., Cooper J. A., Morris R. W. (2015). Sixty-five common genetic variants and prediction of type 2 diabetes. *Diabetes*.

[13] Kursa M. B. (2014). Robustness of Random Forest-based gene selection methods. *BMC Bioinformatics*.

[14] Cai Z., Xu D., Zhang Q., Zhang J., Ngai S.-M., Shao J. (2015). Classification of lung cancer using ensemble-based feature selection and machine learning methods. *Molecular BioSystems*.

[15] Chen Y.-C., Ke W.-C., Chiu H.-W. (2014). Risk classification of cancer survival using ANN with gene expression data from multiple laboratories. *Computers in Biology and Medicine*.

[16] Kong Y., Yu T. (2018). A deep neural network model using random forest to extract feature representation for gene expression data classification. *Scientific Reports*.

[17] Hsieh C.-H., Lu R.-H., Lee N.-H., Chiu W.-T., Hsu M.-H., Li Y.-C. (. J.). (2011). Novel solutions for an old disease: diagnosis of acute appendicitis with random forest, support vector machines, and artificial neural networks. *Surgery*.

[18] Zhang Z., Chen L., Humphries B. (2018). Morphology-based prediction of cancer cell migration using an artificial neural network and a random decision forest. *Integrative Biology*.

[19] Janßen R., Zabel J., von Lukas U., Labrenz M. (2019). An artificial neural network and Random Forest identify glyphosate-impacted brackish communities based on 16S rRNA amplicon MiSeq read counts. *Marine Pollution Bulletin*.

[20] Shafiee M. N., Seedhouse C., Mongan N. (2016). Up-regulation of genes involved in the insulin signalling pathway (*IGF1*, *PTEN* and *IGFBP1*) in the endometrium may link polycystic ovarian syndrome and endometrial cancer. *Molecular and Cellular Endocrinology*.

[21] Owens L. A., Kristensen S. G., Lerner A. (2019). Gene expression in granulosa cells from small antral follicles from women with or without polycystic ovaries. *The Journal of Clinical Endocrinology and Metabolism*.

[22] Johnson W. E., Li C., Rabinovic A. (2007). Adjusting batch effects in microarray expression data using empirical Bayes methods. *Biostatistics*.

[23] Ritchie M. E., Phipson B., Wu D. (2015). *limma* powers differential expression analyses for RNA-sequencing and microarray studies. *Nucleic Acids Research*.

[24] Li W. (2012). Volcano plots in analyzing differential expressions with mRNA microarrays. *Journal of Bioinformatics and Computational Biology*.

[25] Yu G., Wang L.-G., Han Y., He Q.-Y. (2012). clusterProfiler: an R package for comparing biological themes among gene clusters. *OMICS: A Journal of Integrative Biology*.

[26] Walter W., Sánchez-Cabo F., Ricote M. (2015). GOplot: an R package for visually combining expression data with functional analysis. *Bioinformatics*.

[27] Breiman L. (2001). Machine learning, volume 45, number 1- springer link. *Machine Learning*.

[28] Günther F., Fritsch S. (2010). neuralnet: training of neural networks. *The R Journal*.

[29] Robin X., Turck N., Hainard A. (2011). pROC: an open-source package for R and S+ to analyze and compare ROC curves. *BMC Bioinformatics*.

[30] Tabl A. A., Alkhateeb A., ElMaraghy W., Rueda L., Ngom A. (2019). A machine learning approach for identifying gene biomarkers guiding the treatment of breast Cancer. *Frontiers in Genetics*.

[31] Wang D., Li J. R., Zhang Y. H., Chen L., Huang T., Cai Y. D. (2018). Identification of differentially expressed genes between original breast cancer and xenograft using machine learning algorithms. *Genes*.

[32] Wang C., Pu W., Zhao D. (2018). Identification of hyper-methylated tumor suppressor genes-based diagnostic panel for esophageal squamous cell carcinoma (ESCC) in a Chinese Han population. *Frontiers in Genetics*.

[33] Zhang Y., Tseng J. T. C., Lien I. C., Li F., Wu W., Li H. (2020). mRNAsi index: machine learning in mining lung adenocarcinoma stem cell biomarkers. *Genes*.

[34] Meena D. K., Manimekalai D. M., Rethinavalli S. (2015). A novel framework for filtering the PCOS attributes using data mining techniques. *International Journal of Engineering Research & Technology*.

[35] Vikas B., Anuhya B., Bhargav K. S., Sarangi S., Chilla M. (2018). Application of the apriori algorithm for prediction of Polycystic Ovarian Syndrome (PCOS). *Information Systems Design and Intelligent Applications*.

[36] Zhang X. Z., Pang Y. L., Wang X., Li Y. H. (2018). Computational characterization and identification of human polycystic ovary syndrome genes. *Scientific Reports*.

[37] Cheng J. J., Mahalingaiah S. (2019). Data mining polycystic ovary morphology in electronic medical record ultrasound reports. *Fertility Research and Practice*.

[38] Ho C.-H., Chang C.-M., Li H.-Y., Shen H.-Y., Lieu F.-K., Wang P. S.-G. (2020). Dysregulated immunological and metabolic functions discovered by a polygenic integrative analysis for PCOS. *Reproductive BioMedicine Online*.

[39] Jesintha Mary M., Vetrivel U., Munuswamy D., Melanathuru V. (2016). PCOSDB: PolyCystic Ovary Syndrome Database for manually curated disease associated genes. *Bioinformation*.

[40] Joseph S., Barai R. S., Bhujbalrao R., Idicula-Thomas S. (2016). PCOSKB: A KnowledgeBase on genes, diseases, ontology terms and biochemical pathways associated with PolyCystic Ovary Syndrome. *Nucleic Acids Research*.

[41] Domingues T. S., Bonetti T. C., Pimenta D. C. (2019). Proteomic profile of follicular fluid from patients with polycystic ovary syndrome (PCOS) submitted to in vitro fertilization (IVF) compared to oocyte donors. *JBRA Assisted Reproduction*.

[42] Lu C., Liu X., Wang L. (2017). Integrated analyses for genetic markers of polycystic ovary syndrome with 9 case-control studies of gene expression profiles. *Oncotarget*.

[43] Jansen E., Laven J. S. E., Dommerholt H. B. R. (2004). Abnormal gene expression profiles in human ovaries from polycystic ovary syndrome patients. *Molecular Endocrinology*.

[44] Haouzi D., Assou S., Monzo C., Vincens C., Dechaud H., Hamamah S. (2012). Altered gene expression profile in cumulus cells of mature MII oocytes from patients with polycystic ovary syndrome. *Human Reproduction*.

[45] Ouandaogo Z. G., Frydman N., Hesters L. (2012). Differences in transcriptomic profiles of human cumulus cells isolated from oocytes at GV, MI and MII stages after in vivo and in vitro oocyte maturation. *Human Reproduction*.

[46] Liu H., Zeng L., Yang K., Zhang G. (2016). A network pharmacology approach to explore the pharmacological mechanism of xiaoyao powder on anovulatory infertility. *Evidence-based Complementary and Alternative Medicine: Ecam*.

[47] Ambekar A. S., Kelkar D. S., Pinto S. M. (2015). Proteomics of follicular fluid from women with polycystic ovary syndrome suggests molecular defects in follicular development. *The Journal of Clinical Endocrinology & Metabolism*.

[48] Skov V., Glintborg D., Knudsen S. (2007). Reduced expression of nuclear-encoded genes involved in mitochondrial oxidative metabolism in skeletal muscle of insulin-resistant women with polycystic ovary syndrome. *Diabetes*.

[49] Nilsson E., Benrick A., Kokosar M. (2018). Transcriptional and epigenetic changes influencing skeletal muscle metabolism in women with polycystic ovary syndrome. *The Journal of Clinical Endocrinology & Metabolism*.

[50] Qiao J., Wang L., Li R., Zhang X. (2008). Microarray evaluation of endometrial receptivity in Chinese women with polycystic ovary syndrome. *Reproductive Biomedicine Online*.

[51] Xu H., Han Y., Lou J. (2017). PDGFRA, HSD17B4 and HMGB2 are potential therapeutic targets in polycystic ovarian syndrome and breast cancer. *Oncotarget*.

[52] Wood J. R., Nelson-Degrave V. L., Jansen E., McAllister J. M., Mosselman S., Strauss J. F. (2005). Valproate-induced alterations in human theca cell gene expression: clues to the association between valproate use and metabolic side effects. *Physiological Genomics*.

[53] Kenigsberg S., Bentov Y., Chalifa-Caspi V., Potashnik G., Ofir R., Birk O. S. (2009). Gene expression microarray profiles of cumulus cells in lean and overweight-obese polycystic ovary syndrome patients. *Molecular Human Reproduction*.

[54] Wang Z., Gerstein M., Snyder M. (2009). RNA-Seq: a revolutionary tool for transcriptomics. *Nature Reviews Genetics*.

