# Supplementary figures and images for: Establishment and Analysis of a Combined Diagnostic Model of Polycystic Ovary Syndrome with Random Forest and Artificial Neural Network
Source: Biomed Res Int. 2020 Aug 20;2020:2613091. doi: 10.1155/2020/2613091 (PMC7455828; doi:10.1155/2020/2613091)

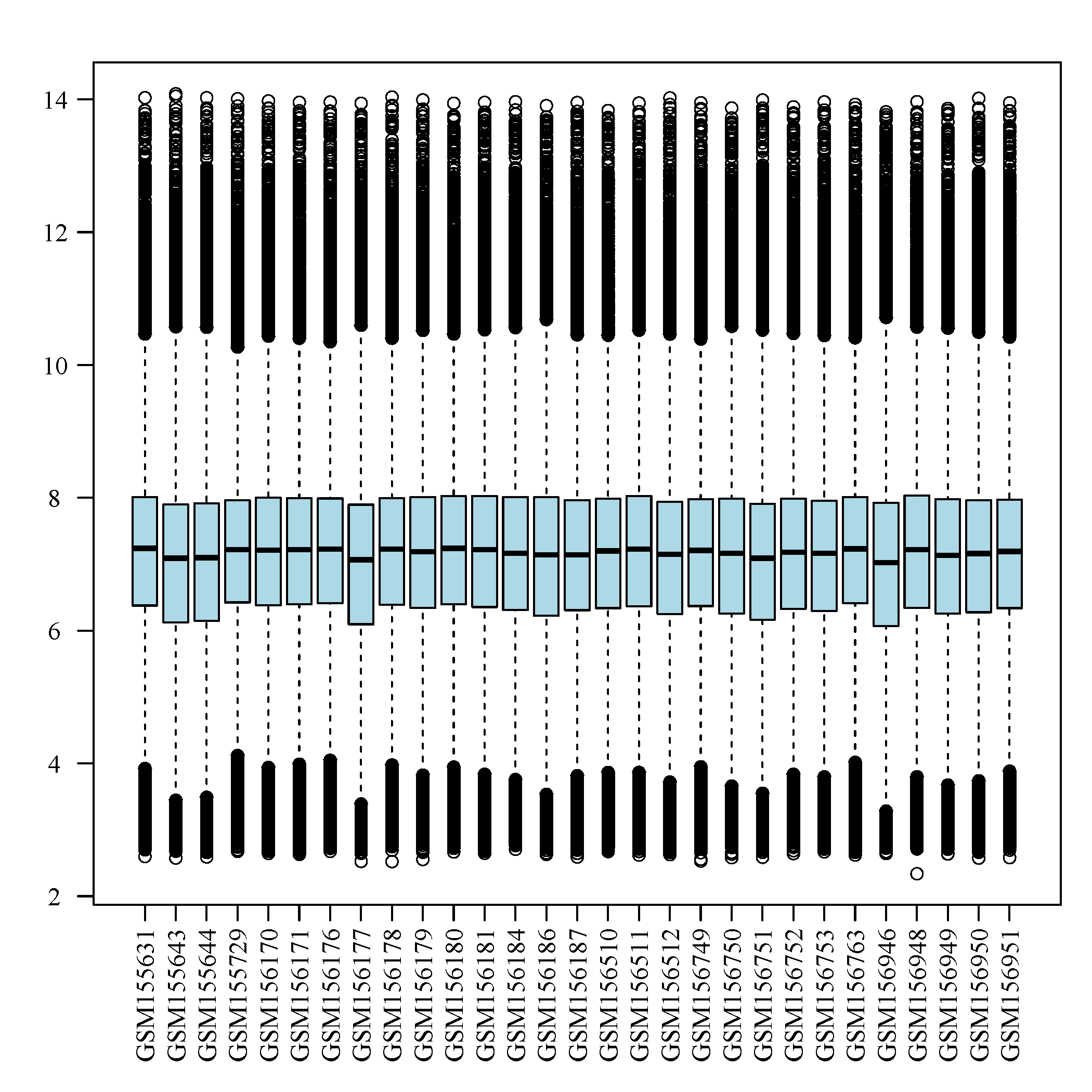

Supplement: Supplementary 1 — Figure S1: boxplot for gene expression data in GSE6798 dataset. The abscissa axis indicates 29 samples in GSE6798 dataset. Axis of ordinates represents gene expression level. [file 2613091.f1.tiff]
